# Effect of Zoledronic Acid on Bone Mineral Density in Men with Prostate Cancer Receiving Gonadotropin-Releasing Hormone Analog

**DOI:** 10.1155/2011/176164

**Published:** 2010-08-19

**Authors:** Anoop Kapoor, Ankur Gupta, Nilay Desai, Hongshik Ahn

**Affiliations:** ^1^Division of Endocrinology, University Hospital, SUNY, Stony Brook and VA Medical Center, Mail Code 111, 79 Middleville Road Northport, NY 11768, USA; ^2^Division of Endocrinology, SUNY, Stony Brook and VA Medical Center, University Hospital, T-060, HSC, Level 15, Stony Brook, NY 11794, USA; ^3^Boice-Willis Clinic, 901 N. Winstead Ave, Rocky Mount, NC 27804, USA; ^4^Department of Applied Mathematics and Statistics, Stony Brook University, Stony Brook, NY 11794-3600, USA

## Abstract

*Background*. Loss of bone density with androgen deprivation therapy for prostate cancer is well recognized. We assessed the effects of quarterly infusion of zoledronic acid on bone mineral density (BMD) and markers of bone turnover over a one-year period in men receiving gonadotropin-releasing hormone analog (GnRH-a) for prostate cancer. *Methods*. 41 subjects were randomly assigned to treatment with zoledronic acid (4 mg) IV infusion or placebo every 3 months. The primary endpoint was the change in the lumbar spine BMD after 12 months of treatment. *Results*. The change in vertebral BMD in the zoledronic acid group (+7.93 ± 1.4%) was significantly (*P* < .05) greater than the change in the placebo group (+0.82 ± 1.7%) as was the change in left femoral neck BMD (+5.05 ± 1.4% for the zoledronic acid group versus −0.48 ± 1.4% for the placebo group). The decrease in biochemical markers of bone turnover was significantly (*P* < .05) greater in the zoledronic acid group compared to the placebo group. *Conclusion*. Quarterly infusion of zoledronic acid for 1 year improved vertebral and left femoral neck BMD with a decrease in bone turnover markers in men on GnRH-a treatment. Zoledronic acid treatment appears to be promising in men with low BMD receiving GnRH-a treatment.

## 1. Introduction

Prostate cancer is the most common visceral malignancy and the second leading cause of death from cancer in men [1]. The dependence of prostate cancer on androgens has led to the use of GnRH-a in the management of prostate cancer [2, 3]. Androgen ablation using GnRH-a suppresses testosterone production [3]. There are several studies showing the effect of GnRH-a treatment on bone mineral density and bone metabolism in men with prostate cancer [4–6]. GnRH-a treatment results in reduction in bone mineral density (BMD) and elevation in biochemical markers of bone turnover. Bisphosphonates decrease bone resorption, primarily by inhibiting osteoclast activity and proliferation [7]. Zoledronic acid, the most potent intravenous bisphosphonate currently available, decreases bone turnover and improves BMD in men [8–12]. We conducted a randomized, double-blind, placebo controlled study to evaluate the effect of zoledronic acid on BMD and biochemical markers of bone turnover in men receiving androgen deprivation therapy for prostate cancer. 

## 2. Materials and Methods

### 2.1. Study Design

The study was a prospective, randomized, double-blinded, placebo controlled trial to determine whether concomitant therapy with zoledronic acid could prevent the progression and/or reverse the bone loss in men with prostate cancer receiving GnRH-a treatment. The primary objective was to evaluate whether such treatment would prevent bone loss in the lumbar spine, and the secondary objective was to evaluate the effects of treatment in the nondominant hip. The subjects were randomly assigned to treatment with zoledronic acid 4 mg IV infusion every 3 months or placebo over a study period of 12 months. All subjects received oral calcium (elemental) 1000 mg daily and vitamin D 500 IU daily. The study was approved by the institutional review board (IRB) at the Northport Veterans Affairs Medical Center.

### 2.2. Inclusion Criteria

Patients with prostate cancer receiving regular injections of gonadotropin-releasing hormone analogs (leuprolide or goserelin acetate) at the VA Medical center in Northport, New York were screened with an initial questionnaire ascertaining duration of therapy with GnRH-a and subsequently underwent an initial BMD measurement. Those patients with evidence of osteopenia or osteoporosis were asked to participate in the study. Osteoporosis was defined as bone mineral density 2.5 standard deviations below the mean (T-score <−2.5) and osteopenia as 1 standard deviation below the mean (T-score <−1.0).

### 2.3. Exclusion Criteria

The exclusion criteria were

- use of medication known to affect bone mineral density or bone metabolism (bisphosphonates, calcitonin, chronic steroid use, or estrogens),
- medical history: disorders of bone metabolism (hyperparathyroidism, Paget's disease, or malabsorption syndrome (intestinal resection, pancreatic insufficiency, or celiac sprue)),
- hypogonadism other than biochemical castration (e.g., congenital or surgical castration),
- evidence of prostate cancer metastasis to bone (clinical, biochemical, or radiological evidence of bone metastasis); bone scan was not performed on all patients prior to inclusion in the study,
- malignancy other than nonmelanoma skin cancer (localized basal cell or squamous cell cancer),
- serum creatinine >1.5 mg/dl,
- age >85 years.

### 2.4. Laboratory Evaluation

The following studies were performed at baseline and at 3, 6, 9, and 12 months after initiation of therapy.

1. Markers of bone resorption: urinary N-telopeptide (testing performed at Quest Laboratories).
2. Markers of bone formation: serum osteocalcin and serum bone-specific alkaline phosphatase (testing performed at Quest Laboratories).
3. The following studies were performed at baseline: serum testosterone, serum intact PTH, serum 1,25 (OH)2 vitamin D, and serum 25 OH vitamin D (testing performed at Quest Laboratories). Serum creatinine was performed at Northport VA Medical Center clinical chemistry Laboratory.

### 2.5. Bone Densitometry

Dual energy X-ray absorptiometry (DXA) was used to measure the bone density of the lumbar spine (L_1_–L_4_), left total hip, and left femoral neck using the Hologic QDR-4500A X-ray Bone Densitometer. Quality control of the machine was performed on a daily basis using a spine phantom as per the guidelines of the company. All scans were performed by the same technician in the Nuclear Medicine Department at the VAMC Northport and reviewed by the same radiologist as well as by the principal investigator.

### 2.6. Sample Size

Sample size was based on comparisons of BMD endpoint data (obtained at one year of followup) between the zoledronic acid and placebo groups. To control for group differences in baseline BMD levels, percent change in BMD from baseline was the statistical outcome variable comparison. Determination of required sample sizes per group was based on a two-sided *t*-test with *α* = 0.05 and a power of 90%. The standard deviation (SD) required for sample size calculations was 0.082, as estimated from the standard errors given by Ringe [13]. A similar estimate of SD, 0.07, was obtained from the standard errors given by Orwoll[14]. The first estimate gives the more conservative results. The research plan called for 30 patients per group to have ample power (>90%) for statistically detecting a group difference. This sample size is obtained based on the assumption that the true difference in the zoledronic acid and placebo group means is 0.07 and the standard deviation of each group is 0.082. In our study, due to slow accrual rates, we closed the study before achieving this target sample size.

### 2.7. Randomization

The patients were screened between August 2003 and March 2008 at the VAMC Northport. 42 patients consented to participate in the study. One patient failed the screening. A random number generator randomly partitioned the remaining 41 patients (via their medical record numbers) into two groups (zoledronic acid and placebo). Each group was identified with a code, known only to the biostatistician and to the pharmacist who was not involved in treating or evaluating the study participants and who was responsible for preparing the active and placebo treatments for the participants.

### 2.8. Statistical Analysis

The randomization of the participants into two groups prevented selection bias and resulted in the two groups being similar with regard to baseline clinical characteristics, including BMD levels. BMD measurements on patients were made at two time points: baseline and after 12 months of treatment. Percent changes of BMD after 12 months of treatment were compared. The changes in each measure (BMD and T-score) between baseline and after all infusions were compared between control and treatment groups. A two-sided significance level of 0.05 was used for a two-sample t-test. The normality assumption was found to be satisfied using Shapiro-Wilk test. The data analysis was undertaken by using SAS statistical software (version 9.1; SAS Inc., Cary).

## 3. Results

### 3.1. Baseline and Followup

21 patients were assigned to the zoledronic acid group, and 20 patients were assigned to the placebo group. 10 patients were excluded in the analysis of BMD (4 in the zoledronic acid group and 6 in the placebo group). In the zoledronic acid group, one patient died due to metastasis from prostate carcinoma, and three patients changed their mind after consenting and withdrew from the study. In the placebo group, one patient died due to metastasis from lung cancer, another patient was diagnosed with prostate cancer metastasis to the spine during the study and four patients did not show up for followup DXA scan within a year after the last infusion. Total 31 patients were included in the analysis of bone density. 17 patients (mean age 74.7 years) received infusions of zoledronic acid and 14 patients (mean age 73.4 years) received placebo. All subjects received 1 gram elemental calcium and 500 units of vitamin D daily. All patients who received at least one infusion were included in the analysis of BMD. Twenty-eight patients received all four infusions.

### 3.2. Bone Mineral Density

**Figure 1:**
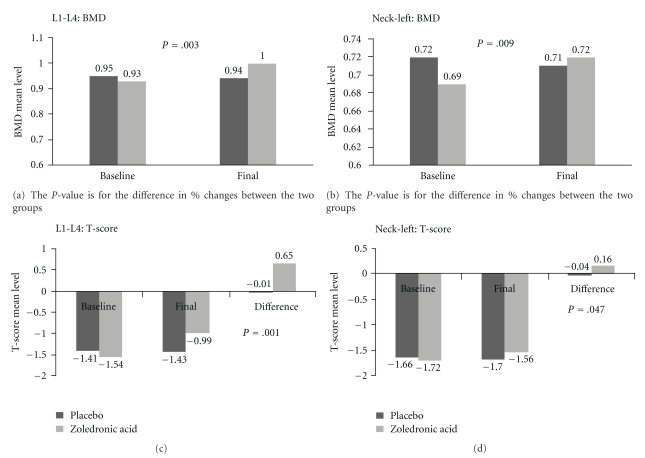
BMD and T-score measures at baseline and final.

There was no significant difference in the baseline BMD measurements between the two groups. The change in vertebral bone density in the zoledronic acid group (+7.93 ± 1.4%) was significantly (*P *<  .05) greater than the change in the placebo group (+0.82 ± 1.7%) as was the change in left femoral neck bone density (+5.05 ± 1.4% for the zoledronic acid group versus −0.48 ± 1.4% for the placebo group). Left total hip BMD increased, 2.27 ± 0.92%, in the zoledronic acid group but this change was not significantly different from the placebo group (Table 1). In the zoledronic acid group, one patient had hardware in the spine, and one patient had left hip replacement. Spine BMD in the patient with hardware in the spine and left hip BMD in the patient with left hip replacement were not included in the analysis. The change in vertebral T-score in the zoledronic acid group (0.65 ± 0.11) was significantly (*P *<  .05) greater than the change in the placebo group (−0.02 ± 0.14) as was the change in left femoral neck T-score (0.16 ± 0.07 for the zoledronic acid group versus −0.04 ± 0.07 for the placebo group) as shown in Figure 1.

### 3.3. Biochemical Markers of Bone Turnover

**Figure 2:**
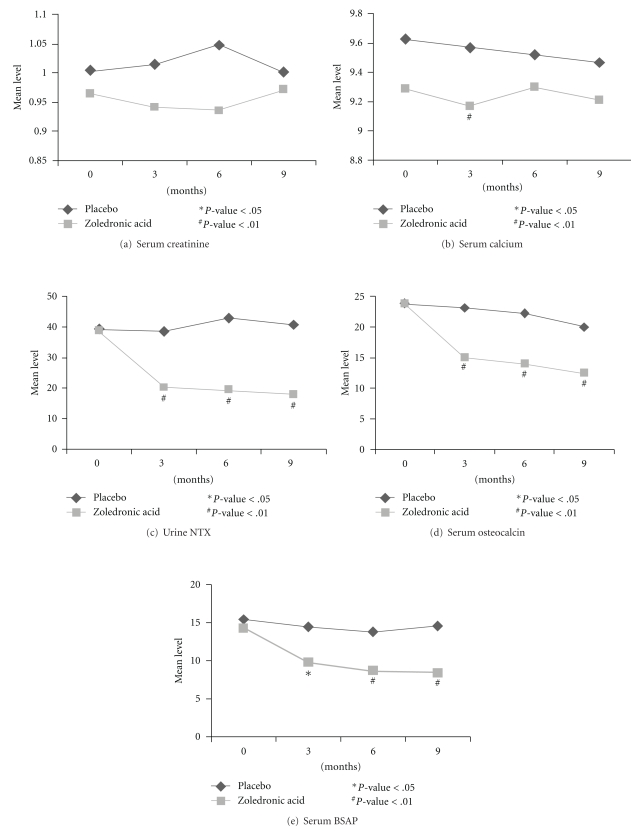
Comparison of biomarker levels between that two groups at each time point.

There were no significant differences in the baseline characteristics (age, serum creatinine, serum calcium, testosterone, parathyroid hormone, 25-hydroxy vitamin D, 1,25-dihydroxy vitamin D, and biochemical markers of bone turnover) between the two groups. All patients in the two groups were hypogonadal with baseline testosterone levels less than 50 ng/dl. The levels of urine N-telopeptide, serum osteocalcin, and serum bone-specific alkaline phosphatase (BSAP) were 49 ± 6.6%, 51.6 ± 6.1%, and 38.8 ± 7.5%, lower than baseline in the zoledronic acid group (Table 2). These changes were significantly lower than the percent changes in placebo at significance level 0.05.  Figure 2 compares the biochemical marker levels between zoledronic acid and placebo groups at each time point. Urine N-telopeptide (NTX), osteocalcin, and BSAP show significant difference between the two groups at 3, 6, and 9 months (*P*-value <  .05).

### 3.4. Adverse Events

Zoledronic acid treatment was well tolerated by most subjects except for one subject who developed atrial fibrillation and another subject who developed reversible acute renal failure in the study. Both subjects received zoledronic acid. There were no cases of osteonecrosis of the jaw.

## 4. Discussion

In our study, zoledronic acid was effective in preventing bone loss and improving markers of bone resorption in prostate cancer patients with low bone mineral density receiving androgen deprivation therapy. The treatment was well tolerated by most of the patients except one subject who developed atrial fibrillation 8 weeks after the first dose of zoledronic acid and another subject who developed acute renal failure following a bout of acute enteritis while also taking ibuprofen. The subject's renal function gradually returned to baseline. These subjects were included in analysis of BMD. The subject with atrial fibrillation was included in analysis of bone biomarkers while the subject with acute renal failure was excluded in the analysis of bone biomarkers.

Our study results are consistent with previous studies showing a beneficial effect of zoledronic acid on bone mineral density done in men with prostate cancer treated with hormone deprivation therapy [8–12]. In our study, we evaluated the effect of quarterly infusion of zoledronic acid on bone mineral density in patients with non-metastatic prostate cancer similar to studies reported by Smith et al. [8] and Ryan et al. [9]. In our study, the change in vertebral bone density in the zoledronic acid group (7.93 ± 1.4%) was significantly (*P *<  .05) greater than the change in the placebo group (0.82 ± 1.7%) as was the change in left femoral neck bone density ((5.05 ± 1.4%) for the zoledronic acid group versus (−0.48 ± 1.4%) for the placebo group). 

Smith et al. [8] reported that mean bone mineral density in the lumbar spine increased by 5.6% in men receiving zoledronic acid and decreased by 2.2% in the placebo group (mean difference 7.8%, *P *<  .001); patients in their study were naïve to androgen deprivation therapy. Ryan and coworkers [9] found that zoledronic acid increased lumbar spine, femoral neck, and total hip BMD yearly by 6.7% (*P *< .0001), 3.6% (*P* =  .0004), and 3.8% (*P *<  .0001), significantly greater than placebo. 

Israeli et al. [10] evaluated the effect of quarterly infusion of zoledronic acid on bone mineral density in patients with locally advanced prostate cancer during the first year of androgen deprivation therapy. At week 52, the least squares mean BMD percentage differences of 6.7% for the lumbar spine and 3.7% for the total hip were significantly greater in the zoledronic acid group in comparison to the placebo group (*P *<  .0001). 

Michaelson et al. [11] and Satoh et al. [12] both evaluated the effect of yearly infusion of zoledronic acid on bone mineral density in patients with prostate cancer. In the Satoh et al. study, patients were naïve to androgen deprivation therapy and had metastases to bone. Michaelson et al. found that the BMD in the lumbar spine increased by 4.0%  ± 1.0% (mean ± SE, *P *<  .001) and femoral neck BMD increased by 2.0% ± 0.6% in men given zoledronic acid (*P* =  .06) whereas Satoh et al. reported that the mean BMD in the lumbar spine increased by 3.5%  ± 0.8% (*P* =  .004) and femoral neck BMD increased by 5.1%  ± 1.3% (*P* =  .0393) yearly in men assigned to zoledronic acid. Both these studies suggest that once-yearly infusion of zoledronic acid might be sufficient in this group of patients [11, 12].

All three biochemical markers of bone turnover (urine N-telopeptide, serum osteocalcin, and serum BSAP) in our study decreased significantly (*P *<  .05) in the zoledronic acid group compared to the placebo group as in previous studies [9–11]. There was significant decrease in serum calcium after the first infusion of zoledronic acid which returned to baseline at subsequent infusions. There was no significant change in serum creatinine level in either group.

Saad et al. [15] studied the effect of zoledronic acid on skeletal complications in prostate cancer patients with bone metastases. In their study, zoledronic acid at 4 mg or 8 mg given every 3 weeks reduced skeletal-related events (defined as pathologic bone fractures (vertebral or nonvertebral), spinal cord compression, bone surgery, radiation therapy to bone, or a change of antineoplastic therapy to treat bone pain) and increased the median time to first skeletal-related event in prostate cancer patients with bone metastases. We did not evaluate skeletal-related events in our study.

Our study has limitations. The sample size was small. We studied only one dosing regimen, and the study duration was only one year. The study was not powered to assess the impact on skeletal-related events. We think studies of longer duration and with different dosing regimens are needed to evaluate long-term safety and efficacy of zoledronic acid in these patients and its impact on skeletal-related events.

## 5. Conclusion

Quarterly infusion of zoledronic acid for 1 year improved vertebral and left femoral neck bone mineral density with a decrease in bone turnover markers in men receiving androgen deprivation therapy with GnRH-a. Our study adds to the previously reported evidence that zoledronic acid should be considered as a treatment option in men with low bone mineral density receiving GnRH-a.

##  Source of Financial Support 

Novartis pharmaceutical company sponsored the study.

## Figures and Tables

**Table 1:** Comparison of mean ± SE percent change in BMD from baseline to final in BMD (gm/cm^2^) between two groups. A two-sided significance level of 0.05 is used for a two-sample *t*-test.

		Placebo	Zoledronic acid	*P*-value
L1-L4 vertebrae	*N*	14	16	**.003 **
	Baseline	0.946 (0.052)	0.928 (0.030)	
	Final	0.944 (0.042)	1.000 (0.031)	
	Mean % change	+0.82 (1.7)	+7.93 (1.4)	

Left total hip	*N*	13	16	.278
	Baseline	0.888 (0.027)	0.868 (0.023)	
	Final	0.893 (0.024)	0.889 (0.029)	
	Mean % change	+0.71 (1.1)	+2.27 (0.92)	

Left femoral neck	*N*	13	16	**.009**
	Baseline	0.718 (0.019)	0.690 (0.029)	
	Final	0.713 (0.017)	0.724 (0.030)	
	Mean % change	−0.48 (1.4)	+5.05 (1.4)	

**Table 2:** Comparison of mean and SE percent change in biochemical markers of bone turnover from baseline to final between the two groups. Significant differences were observed in urine NTX, osteocalcin, and BSAP.

		Placebo	Zoledronic acid	*P*-value
Serum creatinine (mg/dL)	*N*	13	15	.58
	Baseline	0.969 (0.073)	0.960 (0.050)	
	Final	1.008 (0.055)	0.993 (0.055)	
	Mean % change	+9.8 (10)	+3.7 (2.8)	

Serum calcium (mg/dL)	*N*	13	15	.45
	Baseline	9.654 (0.12)	9.267 (0.15)	
	Final	9.515 (0.11)	9.247 (0.11)	
	Mean % change	−1.36 (1.1)	−0.02 (1.4)	

Urine NTX Collagen cross-linked urine (nmol BCE/mmol creatinine)	*N*	13	14	.022*
	Baseline	38.2 (4.9)	41.4 (5.3)	
	Final	40.4 (4.0)	18.8 (2.3)	
	Mean % change	+32 (30)	−49 (6.6)	

Serum osteocalcin (ng/mL)	*N*	13	14	.0003*
	Baseline	23.7 (1.5)	25.2 (2.5)	
	Final	18.6 (1.6)	11.6 (1.4)	
	Mean % change	−22.1 (3.5)	−51.6 (6.1)	

Serum bone-specific alkaline phosphatase (BSAP) (mcg/L)	*N*	13	15	.002*
	Baseline	15.5 (2.0)	14.3 (1.3)	
	Final	14.9 (2.0)	8.1 (0.9)	
	Mean % change	−2.0 (7.3)	−38.8 (7.5)	

*Significant at level 0.05. Standard error follows mean in parentheses.
